# Subjective Well-Being and Its Intrinsic and Extrinsic Motivational Correlates in High Performance Executives: A Study in Chilean Managers Empirically Revisiting the Bifactor Model

**DOI:** 10.3390/ijerph18158082

**Published:** 2021-07-30

**Authors:** Daniela Pradenas, Juan Carlos Oyanedel, Silvia da Costa, Andrés Rubio, Dario Páez

**Affiliations:** 1 Facultad de Psicología y Psicopedagogía, Pontificia Universidad Católica Argentina, Buenos Aires C1107, Argentina; danielapradenas@uca.edu.ar; 2 Facultad de Educación y Ciencias Sociales, Universidad Andres Bello, Santiago 7591538, Chile; juan.oyanedel@unab.cl (J.C.O.); dario.paez@ehu.es (D.P.); 3 Facultad de Psicología, Universidad de Granada, 18011 Granada, Spain; silviadcd2021@ugr.es; 4 Facultad de Economía y Negocios, Universidad Andres Bello, Santiago 7520404, Chile; 5 Facultad de Psicología, Universidad Diego Portales, Santiago 8370076, Chile; 6 Facultad de Psicología, Universidad del País Vasco, 20018 San Sebastián, Spain

**Keywords:** well-being, work satisfaction, extrinsic motivation, intrinsic motivation

## Abstract

This study analyzes the relationship between work satisfaction, family satisfaction, and general well-being in high performance managers in Santiago, Chile. The importance of the satisfaction of intrinsic and extrinsic needs and motivations was examined to advance in the development of a positive organizational psychology, which investigates the factors that reinforce well-being. Seventy-five executives from large and medium-sized companies were surveyed and 8 in-depth interviews were carried out. The main predictors of well-being are, from family satisfaction, the family’s ability to cope with stress and, from work satisfaction, extrinsic aspects such as material conditions of the job and stability, and intrinsic aspects such as recognition and the ability to organize one’s own work. The more general regression model shows that extrinsic job and family satisfaction predict general well-being, not intrinsic satisfaction. The results are discussed in the framework of classical models of motivation, such as Herzberg’s, their relationship to Deci and Ryan’s self-determination theory, and the current study of well-being in organizations.

## 1. Introduction

Life satisfaction, or the cognitive dimension of subjective well-being, is a hot topic in the study of organizations. The importance of the satisfaction of intrinsic and extrinsic needs and motivations was examined as a way to advance the development of positive organizational psychology, which explores the factors that reinforce well-being. In general, research seeks to understand both the causes and the possible effects of well-being (higher productivity, greater job attachment, greater creativity [1]). However, the available evidence regarding these phenomena in the case of company managers is much more limited. This study seeks to take a first look at this phenomenon by means of a study on the relationship between satisfaction with extrinsic and intrinsic aspects in the work and family environment and general well-being in high-performance managers in Santiago, Chile. The relevance of the study of managers is high since they influence different aspects and areas of the organization. In other words, they influence the definition of the company’s strategy, planning, as well as the decision-making process, with medium and long-term scopes. Managers occupy positions that are part of the top hierarchy of their respective organizations, which gives them authority and a high degree of responsibility. In addition to the above, they present important levels of autonomy, belonging, competence, and are usually a difficult group to access [2].

Although there are multiple definitions, the following functional elements characterize high-performance managers: (a) they have responsibility for the organization’s resources and personnel; (b) they report only to the highest levels of the organization; and (c) they plan and organize their work and that of others. In this sense, managers have a controlling role over the organization’s productive process and over people. Managers organize, lead, and build work teams. They are also the ones who make the fulfillment of the organizational mission viable and provide support and sustainability to the teams [3].

The study of high-performance managers has been marked by two main trends. The first is focused on the personal characteristics of the manager, which gives support, in general terms, to the study and intervention in leadership and coaching. The assumption in this line is that, by providing leaders with methods of organizational analysis and self-analysis, they would be able to achieve better results, guiding the members of their respective teams. The second trend, coming from satisfaction studies and positive psychology, points out that the organization is not only concerned with production, but also with providing well-being or happiness to its members. Likewise, in this variant the focus is not only on the organization’s interests, but also on how to articulate the interests of employees (now known as “collaborators”) with the organizational mission. This line has given rise to the emergence of new organizational forms such as “welfare officers”, whose function is to facilitate such articulation of interests [4].

However, in both models, the manager is understood as a mediator between the organization’s mission and the motivation and control of employees. This has led to the fact that little is known about managers, except for some limited efforts from sociology, referring to the low mobility among group members, the existence of the so-called “social closures” or barriers to entry into the group. A high percentage of top executives in Chile, where the empirical study was conducted, belong to a high-status socioeconomic group and come from a limited group of elite educational establishments [5,6], and their occupational success would not necessarily be associated with scholastic merit [7,8]. This study will examine well-being and its correlates in the case of these managers.

### Well-Being and Its Factors in Organizations

Subjective well-being as a subject of study is approximately 30 years old and is still a developing topic in the social sciences. It has also been an important part of the debate in public policy. The publication of the World Happiness Report [9] and the Better Life Initiative [10] reflect the concern about the topic in international organizations. Subjective well-being, as a concept, usually considers three associated dimensions: positive affect, negative affect, and life satisfaction [11]. Life satisfaction is understood as an individual’s overall assessment of the quality of his or her life in the face of his or her circumstances [12]. This can be global [13], or focused on specific domains: personal relationships, achievement, assets, health, etc. [14,15]. Subjective well-being is associated with a number of positive elements: lower prevalence of illness [16,17], better mental health [18,19], higher life expectancy [20], greater creativity [21], and higher work productivity [22,23].

A specific area of well-being is work satisfaction, understood as “a pleasant or positive emotional state resulting from the appraisal of work or work experiences and as a function of the perceived relationship between what one wants from one’s work and what one perceives it offers” [24]. In this sense, work satisfaction would be intrinsically related to the expectations one has with respect to work-for example, with respect to the “psychological contract”, referring to the role and expectations that people have in the organization [25].

To understand what elements affect work satisfaction, classical studies turned to motivation theorists, beginning with content-based theories, such as those based on universal psychological needs, particularly, Maslow, who in his hierarchy of needs theory points out that people vary their motivational elements progressively, first satisfying basic needs, then social needs, and finally personal growth or self-actualization [26]. Although still very popular today in the applied field, this theory has not been fully endorsed by studies, as different needs (e.g., safety and self-actualization) can be present simultaneously [27] (although there have been defenses of Maslow’s theories [28]). Other content-based theories have focused on the differences between individuals on how they establish their motives [29]. Personality traits have been used to explain these differences [30], while more recent research has focused on core self-evaluations as a construct capable of predicting effort, persistence, and work outcomes [31,32]. On the other hand, “context-oriented theories focus on features of the environment that affect motivation and performance via their provision of affordances and constraints for motive satisfaction” [29]. Among them, Herzberg’s two-factor theory approached motivation from the existence of two groups of elements associated with work: hygienic factors, which are those that surround people and are beyond their control, and motivating factors, which are related to what people can control [33]. Further theories on motivation have focused on the subjacent processes, such as self-regulation and ego depletion [34,35]. For the purposes of the present study, two approaches to this phenomenon will be considered: Herzberg’s two-factor theory [33], and Deci and Ryan’s self-determination theory [36].

According to Herzberg’s theory, the former refers to working conditions in the broadest sense, such as salary, company policies, physical environment, safety at work, etc. According to the bifactor model, these factors can only prevent job dissatisfaction or avoid it when it exists, but they cannot determine satisfaction, since this would be determined by the motivating factors, which would be those that are inherent to the job: job content, responsibility, achievement, etc. Several studies support Herzberg’s idea that the factors that lead to work satisfaction are different from, and not simply opposed to, the factors that lead to job dissatisfaction [37]. However, from a methodological point of view, it has been argued that hygiene motivation theory is methodologically constrained, as it only produces supportive results if Herzberg’s technique of critical incident interviews of satisfaction and dissatisfaction is used, and that the use of different methods produces different results [24]. When different data collection methods have been used, they have failed to produce results that were consistent with Herzberg’s results [24,27]. On the other hand, the question of the generality and validity of the findings has been raised, arguing that the use of a single measurement method (semi-structured interview) was not sufficient to ensure general validity. It has also been raised that it is a theory that applies to “white-collar” workers [27]. Finally, the idea that motivators and hygiene factors are two independent factors and that the latter factors have no impact on motivating employees has been criticized [38,39]. It has been argued that some hygiene factors can act as motivators, and motivators can serve as sources of dissatisfaction and satisfaction. This idea was supported by several investigations [24]. It is now accepted that hygiene factors, such as money or safety, are motivators, although of an extrinsic nature—hence the term intrinsic and extrinsic motivators, which Herzberg called motivators and hygiene factors, respectively [40].

Currently, one of the most widely accepted and developed theories of needs is that of Deci and Ryan [36]. These authors differentiate between intrinsic and extrinsic motivation: “the most basic distinction is between intrinsic motivation, which refers to doing something because it is inherently interesting or enjoyable, and extrinsic motivation, which refers to doing something because it leads to a separable outcome” [36] (p. 55). When extrinsically motivated, workers do something in order to receive something of value to them, such as a promotion, a pay increase or a bonus, or to avoid something negative such as a demotion or dismissal. Intrinsic motivation comes from performing the task, not the consequences of performing it. Employees do their job because the tasks they do at work are enjoyable, meaningful, and interesting [41]. In general, intrinsic motivation is more strongly associated with well-being, performance, and creativity than extrinsic motivation—although the latter is not negatively associated with positive outcomes [42]. Deci and Ryan identify as motivations: (a) belongingness; (b) competence and self-esteem; and (c) autonomy or self-determination. Self-determination theory posits that the satisfaction of three basic psychological needs leads to high well-being. These are: (a) autonomy, a sense of agency, choice, and authenticity about thoughts and behaviors; (b) competence, referring to a sense of efficacy and self-esteem, and a sense that one can have a meaningful impact on one’s environment; and (c) belonging relationships, feeling that others care about one’s self and feeling close to others [43,44,45,46]. These authors posit that people possess needs that can be met by the organization, fostering their well-being, through the satisfaction of these needs.

Motivation theories reviewed and Herzberg items (see Table 1) can be organized and related to their indicators [47]. Items related to Responsibilities, Achievements, Growth, Promotion, Recognition and Work itself categorized under Herzberg’s motivation factors are similar with Maslow’s Self Actualization and Self Esteem, and Deci & Ryan Autonomy and Competence. The indicators of these needs would be items linked to recognition, status, progress and achievements, as well as items linked to personal growth, responsibility, challenging work and freedom. Relationship with Peers, Personal Life and Supervision related items categorized under Herzberg’s hygiene factors are congruent with Maslow’s Belongingness and Deci & Ryan relatedness need. The indicators of this need would be items related to the quality of interpersonal relationships and to the satisfaction of personnel policies. Pay and Benefit, Work Condition, Job Security and Company Policy and Administration are congruent with Maslow’s Safety and Physiological Need. Related to these needs are items linked to working conditions, job security, salary, and quality of life (see Table 1 for a synthesis).

The following table presents an organization of the motivation theories reviewed and the indicators of these theories.

Despite criticisms and discussions, studies in the workplace have confirmed the existence of two factors or dimensions of well-being at work, which partly overlap with Herzberg’s ideas. These have been recovered from the field of positive psychology [33]. Of particular relevance is the approach of Warr [48] whose scale will be used in the present research [49]. A confirmatory factor analysis of work-related well-being data carried out by Daniels [50] found a factor solution with multiple first-order dimensions and two second-order factors corresponding to negative and positive affect as the best model of work-related affective well-being. In reference to subjective well-being, negative affect seems to be related to neuroticism, self-reported stress, health problems, frequency of unpleasant events, and avoidance coping. Meanwhile, positive affect correlates with extraversion, sociability, frequency of pleasant events and approach coping [51,52]. An important correlate of positive affect is social relationships and in particular family satisfaction, which partly covers the relational need described above. Family satisfaction refers to how family members feel about their levels of cohesion and flexibility. Higher levels in these two dimensions would imply a higher bonding or commitment of the family members, which would allow them to cope with a greater number of situations [53]. It should be noted that satisfaction with the family is associated with well-being with the same intensity in all cultures, suggesting that it is a universal factor of well-being [54]. The evidence indicates the existence of relevant associations between family satisfaction (relational need in part), extrinsic work satisfaction (security, salary), intrinsic work satisfaction (competence and self-determination), and general well-being or life satisfaction. Based on the literature reviewed and considering the sample—high-income executives—we expect intrinsic elements to outweigh extrinsic elements in terms of work satisfaction [24,55].

## 2. Materials and Methods

### 2.1. Participants and Procedure

Convenience sampling was carried out, since its formulation is ideal for this population, as it allows specific contact with clearly delimited populations such as this one. For this purpose, links were carried out with business organizations, in addition to recruiting participants according to snowball sampling. Eighty-two managers participated in the survey, although for the purposes of the analysis only the proportion of them who responded to the survey in full (*n* = 75) was used. The sample was 75% male, with a mean age of 45 years (range between 35 and 69).

Participants had to log into the Surveymonkey platform and answer a self-administered questionnaire. Participants were contacted in two phases: first, a direct invitation was sent via email and, subsequently, through a generic link that was sent through a business organization (Foro de Comunicación Corporativa, FOCCO). The survey was administered over a period of two months, from November to December 2017.

### 2.2. Instruments

All the scales used range from 0 to 10, where 0 means “totally dissatisfied” and 10 means “totally satisfied”.

#### 2.2.1. Personal Wellbeing Index (PWI)

This index inquires about general life satisfaction by analyzing satisfaction with specific areas of life. International studies show four main dimensions that are significant in relation to standard of living, health, achievements, and personal relationships. In the case of the present study, the 7-item version was used (item 8 refers to religious beliefs). Cronbach’s Alpha for this scale was *α* = 0.86 for the 7 items, which is consistent with previous studies in other Chilean populations [56]. Items of the scale are presented in Table A1 in the Appendix A.

#### 2.2.2. Work Satisfaction Scale

We used the version validated in Spanish by Pérez-Bilbao and Fidalgo [57,58], based on the Herzberg model. It is composed of 15 items, generating two subscales, one referring to intrinsic satisfaction and the other to extrinsic satisfaction. For the purposes of this study, one item was modified and one item referring to workload was added. The items are presented in Table A2 in the Appendix A. Items 1, 3, 5, 7, 9, 10, 12, 14, and 16 are extrinsic, and the others are intrinsic. A factor analysis confirms that two factors are found, one intrinsic and one extrinsic. However, both the overall scale (sixteen items, *α* = 0.926) and the subscales of extrinsic satisfaction (nine items, *α* = 0.879) and intrinsic satisfaction (seven items, *α* = 0.878), present high internal consistency.

#### 2.2.3. Family Satisfaction Scale

This scale evaluates the degree of satisfaction with ten aspects of family dynamics, five of which are related to cohesion and five to adaptability. It is based on Olson’s [59] circular model, in which these two aspects are evaluated jointly, providing flexibility to the model. The brief 10-item version was used for this study (Items are presented in Table A3 in the Appendix A). The scale presents an α = 0.928, so it has acceptable properties for use in the present study.

### 2.3. Data Analysis

Internal consistency was contrasted using Cronbach’s coefficient. To characterize the sample, descriptive and exploratory factor analyses were carried out, as well as a comparison of means. To examine the hypotheses, the factors of job and family satisfaction were correlated with well-being. Multiple regressions were used to examine which items best predicted subjective well-being.

An exploratory factor analysis using all scale’s item found five factors, rejecting the existence a unique common factor. Following the suggestion of an anonymous reviewer, an exploratory factor analysis (EFA) was carried out using the items of all scales–maximum likelihood method and varimax rotation. KMO (above 0.5 and value of 0.83) and Bartlett’s test (χ2 (561) = 2091, *p* = 0.0001) were satisfactory. Seven factors were found. However, five factors explain 66% of variance and a scree test also suggest that five factors are sufficient to fit the data. The first factor explains 20% of variance and all items of the SWF load in this dimension. The second factor explains 18% of variance and seven items of intrinsic satisfaction with job load in this dimension—but also four items extrinsic satisfaction with job related to relations between manager and employees, as well as job security. The third dimension explains 11.22% of variance and four extrinsic satisfaction items loads in this dimension—but also satisfaction with personal relations, health, and community and one intrinsic satisfaction with job item. The fourth dimension explains 11.20% of variance and four PWI items load in this dimension. The fifth dimension explains 6.4% of variance and two extrinsic satisfaction with job items (working hours and salary) and a SWF item (time spent with family) load in this dimension. This EFA rejects the existence on a unique common factor and reproduce a dimension for SWF, for PWI and two different dimensions of satisfaction with work. However, as usual with EFA, items show cross factors load to some extent and some items load in some dimensions reflecting the specific experience of the sample. For instance, a more favorable work schedule is associated with greater possibilities of spending time with the family and the salary helps family activities to be satisfactory, as reflected in the fifth factor. Given the small sample size and the low ratio between items and observations, these deviations from what is expected from the items of the scales are acceptable. See Factor Analysis in Table A4 in the Appendix A.

## 3. Results

### 3.1. Descriptives of the General Well-Being, Job Satisfaction, and Family Satisfaction Scales

Descriptive data by item for the general well-being scale, the job satisfaction scale, and family satisfaction in Table 2 (for item-by-item measures, see Table A1, Table A2 and Table A3 in the Appendix A).

The highest dimensions of managerial well-being are those related to achievement and standard of living. The lowest are those related to feeling part of their community and future security.

An exploratory principal components factor analysis, varimax rotation, imposing two dimensions found one intrinsic and one extrinsic factor, although the extrinsic item of job stability weighed on the intrinsic factor, and the intrinsic item of recognition weighed on both factors.

The highest mean on the job satisfaction scale corresponds to the intrinsic satisfaction scale, and to the items on responsibility assigned to him/her, and variety of activities he/she carries out at work.

The highest means correspond to the items on the degree of closeness among family members, the family’s ability to share positive experiences, and mutual concern among family members.

### 3.2. Relationships between Subjective Well-Being, Job and Family Satisfaction, and Socio-Demographic Variables

When analyzing differences by sex (see Table 3), using analysis of variance, women report higher family satisfaction and subjective well-being, while men have higher job satisfaction, although the differences are not significant—all *F*(1, 73) < 1.73, *p* = 0.245.

When analyzing the differences in well-being and satisfaction by sociodemographic variables (see Table 4), results show that people working in small companies (less than 10 people) have the highest levels of satisfaction and well-being in the sample, although the differences are not significant—all *F*(1, 73) < 1.40, *p* = 0.250.

When analyzing the information on the basis of marital status (see Table 5), single people report higher levels of satisfaction and well-being. It is the divorced/separated who report lower satisfaction in all areas, although the differences are not significant—all *F*(1, 73) < 1.26, *p* = 0.290.

### 3.3. Correlations between Dimensions of Job Satisfaction, Family Satisfaction, and Well-Being

Correlations between the different variables were carried out (see Table 6). A strong correlation was found between subjective well-being and family satisfaction, intrinsic and extrinsic job satisfaction. The main association is with extrinsic satisfaction. It should be noted that the association between extrinsic and intrinsic job satisfaction is greater than 0.70, suggesting that they form a single construct. A paired t-test comparison of means showed that there was no difference between total and family job satisfaction. In contrast, intrinsic job satisfaction was higher (*M* = 8.18, *SD* = 1.23) than extrinsic job satisfaction (*M* = 7.71, *SD* = 1.48), *t*(74) = 5.01, *p* = 0.0001.

### 3.4. Multiple Regression of General Well-Being on Job and Family Satisfaction

To examine which dimension is more important as a predictor of managers’ subjective well-being or life satisfaction, a linear regression was applied. Personal Well-Being Index was used as the dependent variable, and the components of the Job Satisfaction Scale (model 1) and the Family Satisfaction Scale (model 2) as independent variables. Finally, an integrated model was used with both scales as predictors of the Personal Well-Being Index (model 3). We carried out a multiple regression using family satisfaction as predictors, then adding total extrinsic and intrinsic job satisfaction. The model including family satisfaction and intrinsic job satisfaction showed an R-squared of 0.36, *F*(2, 72) = 21.68, *p* < 0.0001 with significant standardized betas of B = 0.33 and B = 0.32, respectively. The model including family satisfaction and extrinsic job satisfaction showed an R-squared of 0.44, *F*(2, 72) = 30.62, *p* < 0.0001 with significant standardized betas of B = 0.27 and B = 0.38 respectively. When including the three predictors, an R-squared of 0.44, *F*(3, 71) = 20.62, *p* < 0.0001 was found with significant standardized betas of family satisfaction of B = 0.28 and extrinsic job satisfaction of B = 0.41, while intrinsic job satisfaction obtained a non-significant beta of −0.06, whose negative character is explained by the strong multicollinearity with extrinsic satisfaction.

To explore more precisely the predictors of personal well-being, the regressions were repeated, this time using the specific items of the dimensions. The first resulting model (which integrates as predictors the items of the two job satisfaction dimensions, see Table 7) explains 50.4% of the variance and is significant (*F* = 5.695; *p* < 0.0001). The significant predictors of the model are satisfaction with the physical conditions of the job (*β* = 0.298; *p* < 0.05), which corresponds to extrinsic satisfaction, while the other three predictors respond to intrinsic satisfaction: freedom to choose your work method (*β* = 0.284; *p* < 0.05), influence you have in the company (*β* = −0.324; *p* < 0.05), and the recognition you get for a job well done (*β* = 0.476; *p* < 0.05).

Model 2 (see Table 8) explains 38.2% of the variance and is significant (*F* = 5.576; *p* < 0.000). The significant predictors of the model are: family ability to cope with stress (*β* = 0.597; *p* < 0.005) and family ability to share positive experiences (*β* = −0.635; *p* < 0.005), which has a negative coefficient.

In the case of model 3 (see Table 9), we chose to use a hierarchical and automatic adjustment model in order to simplify its interpretation. The resulting model, after 20 iterations, explains 61.5% of the variance and is significant (*F* = 17.878; *p* < 0.0001). The significant predictors of the model address the three dimensions under study: on the one hand, the appraisal of their family’s abilities to cope with stress (*β* = 0.512; *p* < 0.001) and to share positive experiences (*β* = −0.283; *p* < 0.05). On the other hand, extrinsic dimensions of work, physical conditions (*β* = 0.278; *p* < 0.005), and job stability (*β* = 0.215; *p* < 0.05); and intrinsic dimensions: freedom to choose their work method (*β* = 0.240; *p* < 0.01), recognition obtained for work well done (*β* = 0.224; *p* < 0.05), and influence they have in the company (*β* = −0.232; *p* < 0.05).

### 3.5. Interviews on Satisfaction with Work and Life in General

Interviews were conducted to examine qualitatively how high-performing managers perceived their life and well-being. For most of the eight interviewees, work is central to their perception of themselves, as the following two excerpts exemplify:

“It’s super relevant. I feel comfortable working. The work mobilizes me, I like what I do. In geology it’s particularly important to like what you do.” (Male, 52 years old, single, no children).

“It is fundamental. It’s not an accessory, it’s not that Monday to Friday I’m in a place and that’s it. It defines me by the amount of time it takes up in my life, by what I’m passionate about, by what I like” (Male, 45 years old, married, 3 children).

Faced with the question “how satisfied are you with life?” the responses are mostly very positive, as exemplified by excerpts from five of the eight interviews:

“I am very satisfied with my life because this has been a year of many changes (...) I would say that it has been a very positive change, which has given me a lot of family, personal and professional stability” (Male, 35 years old, married, 2 children).

“I am very satisfied with my life. In terms of work, I do what I like to do in a quiet place, and the levels of demand are what I like. The subjects I see I love, perfect schedules, income not so good, but you can’t have everything in life” (Female, 46 years old, married, 4 children).

“I am fully satisfied with my life, my family, my children, my husband, my job, with all the inconveniences that life itself has, but yes, I am satisfied” (Female, 42 years old, married, 2 children).

“Very satisfied with my life. Content. It makes me content to have achieved the difficult work-life balance” (Female, 37 years old, married, 1 child).

“Satisfied on all levels, family, love, intellectual, sports. In all aspects” (Male, 42 years old, married, 1 child).

It is possible to see in a couple of cases—where managers have been working for many years and in areas such as mining, with a shift system—that people tend to have a lower level of life satisfaction, because they have sacrificed time with their families for work reasons:

“In general I don’t feel very satisfied with my life, there is a big imbalance between professional and personal. Clearly, work life is easier than personal life. That must be a sum of things (...) I think that the particular fact of the profession generates double or triple challenges, basically being away from the family. Of the 23 years I have been working, 21 have been away from home” (Male, 52 years old, married, 3 children).

This gives rise to the idea that, in these cases, the valuation of the family diminishes life satisfaction precisely because of this loss of common experiences. In order to show the relevance of the gender issue, it is important to point out that some women—despite the position they hold and being recognized as high-performance managers—recognize that they have had to postpone their work for the benefit of the family:

“My family is very important to me, much more important than work. I could have had a more successful career, in professional terms, but I preferred to be with my family and see my children happy. I went through a tremendous mourning at the beginning of my life, when one realizes that in reality one cannot aspire to such high positions when one cares about one’s family” (Female, 46 years old, married, 4 children).

The main attributes of their organizations mentioned by the high-performing managers interviewed are summarized in Table 10:

## 4. Discussion

Most of the sample shows high levels of life satisfaction. However, there is a higher prevalence of job satisfaction over family satisfaction in the conceptualization of life satisfaction. The highest dimensions of managerial well-being are those related to achievements and standard of living. The lowest are those referring to feeling part of their community and future security—although in both cases these are scores above 7 on the 0 to 10 scale. The relatively low scores for “feeling part of society” could be interpreted in relation to the fact that high-performing managers are a small group, difficult to reach because they are at the top of the organizational hierarchy. In addition, it could be influenced by their responsibility in decision-making processes, which would generate what is known as “leader loneliness” or “command loneliness”. The relatively low score on “future security” could be explained by the fact that these managers are familiar with the internal maelstrom of the organization’s processes and know that they are facing high levels of competition, since their positions are well remunerated and there are many potential candidates, and they tend to be associated with performance goals.

The intrinsic and extrinsic dimensions of job satisfaction are highly valued by the sample. Intrinsic aspects, such as autonomy and job recognition, scored higher (8.2) than extrinsic aspects (7.6)—although the difference is of degree and the evaluation is very positive in general. However, although both dimensions showed high internal consistency, they also showed a strong association, which is compatible with a single common factor.

Job satisfaction is an important predictor of general well-being, and a stronger predictor than family satisfaction. The qualitative interviews corroborate that for most of the sample, work is fundamental to their well-being. Of the components of the job satisfaction scale that predict general well-being, three predictors correspond to intrinsic satisfaction: (a) freedom to choose one’s work method, which is linked to the need for autonomy; (b) the recognition obtained for a job well done, linked to the need for competence; and (c) the influence one has in the organization, which is linked to the need for esteem and competence. In the case of the intrinsic item of influence, a negative coefficient is obtained. This may be related to the fact that, in the case of managers, influence is associated with responsibility, which implies a higher psychological burden. Finally, satisfaction with the physical conditions of the job, which corresponds to extrinsic satisfaction, also predicts well-being. According to the literature, in the case of job satisfaction, intrinsic rather than extrinsic elements are assumed to be more important [36,55]. This is partially supported in the analysis using dimension items as predictors, since there are more significant intrinsic than extrinsic betas, and the former are of higher size than the latter. Thus, recognition of competence and autonomy would be constitutive factors of managerial identity and well-being.

However, the regression that considered the items of the three dimensions under study showed that the extrinsic items factor predicted general well-being or life satisfaction with a similar weight to the extrinsic items, as well as that family satisfaction played a role. Valuing their family’s ability to cope with stress (with the highest beta of all) and sharing positive experiences (with negative beta and lower beta similar to the other coefficients) predicted well-being in the last regression model. On the other hand, two items from the extrinsic dimension of work, such as physical conditions and job stability, predicted well-being with medium high betas. Finally, three intrinsic items showed medium significant betas: the freedom to choose one’s work method, the recognition one gets for a job well done, and the influence one has on the company.

The hypothesis that the intrinsic factor is more relevant than the extrinsic factor is only partially confirmed in the more global analysis, since the extrinsic betas (stability and physical conditions) are of similar size to the intrinsic ones (freedom and recognition). Further, the relational-familial item presents the highest weight. In addition, it should be noted that when the totals for family satisfaction, extrinsic and intrinsic job satisfaction were used, only the first two items significantly predicted overall well-being. These results suggest that basic security and relational needs are as important as autonomy and competence needs for the well-being of our subjects, which is consistent with the coexistence of basic, relational, competence, and self-determination needs [27,36], or the coexistence in the same people of post-materialistic values of personal development and materialistic values of security and stability [60]. The exploratory nature of the final model can be highlighted, since the hierarchical model is criticized for being “opportunistically” adapted to the sample and not useful for contrasting hypotheses; for this purpose the method of entering all variables simultaneously is used [61].

On the other hand, family satisfaction predicts, albeit to a lesser extent, overall well-being. In particular, the component of the family’s ability to cope with stress is an important source of stress—it has the highest beta in the model that integrates family and work predictors. Let us note that the family’s ability to share positive experiences has a negative coefficient on well-being. This situation may be due to the scarce time that managers have, so that high satisfaction with this variable may mean lower life satisfaction as more time is devoted to being with the family and less to work. In the interviews, two people presented a lower level of life satisfaction, since for work reasons they have sacrificed time with the family. The appreciation of the family, in particular the sharing of positive experiences, may decrease life satisfaction, precisely because work implies not having these experiences in common. This coefficient may be due to multi-collinearity with the other family item. We must ask ourselves whether the same does not occur with the influence on the company—although in both cases, as has been argued, there may be a real logic behind this negative coefficient.

As an explanation for the prevalence of job satisfaction over family satisfaction in general well-being, it emerges from the interviews that most of the participants indicate that they are satisfied with their families, because they perceive that their families are “doing well”. However, when faced with the possibility that the family might lose its status or attribute of “being well”, the majority indicated that it would affect their relationship with work. This is confirmed by the fact that they stated that they can dedicate themselves to work—with the depth or intensity that they do on a daily basis—as a consequence of having the issues of home and family resolved, so that the family would be a protective element of life satisfaction in this condition.

This study is a pioneer in providing information on subjective well-being and its correlates in high-performing executives. The evidence was obtained both through a quantitative survey and qualitative interviews. Among the limitations, it should be noted that the sample is not very large, so it would be convenient to work with larger samples in future studies, although this is a difficult group to access. Another limitation of the study is that it is cross-sectional, so that appeals to causal relationships are not possible, and it relies solely on self-reports. Finally, another limitation is that it shares a form of measurement, and the content of the items share the reference to satisfaction—in general that of well-being and with specific facets in the other two scales. It is likely that the high variance explained is due to the use of similar measurements and with relatively similar contents. Considering the above, future lines of research should consider larger samples, longitudinal designs, and variables that overlap each other as little as possible.

## 5. Conclusions

This study considers an initial investigation, which provides evidence in a field not previously addressed, in a population with difficult access. The most important results include that the main predictors of well-being for the high-performance executives of the sample are, from family satisfaction, the family’s ability to cope with stress, and, from work satisfaction, extrinsic aspects such as material conditions of the job and stability, as well as intrinsic aspects such as recognition and the ability to organize one’s own work. Furthermore, the results showed that extrinsic job and family satisfaction predict general well-being, not intrinsic satisfaction. Future research should consider larger samples (considering the challenge this presents, given the access problems mentioned) and longitudinal designs (allowing the identification of possible causal effects), in order to have more evidence to understand the research problem, considering the relevance of this for positive organizational psychology.

## Figures and Tables

**Table 1:** Needs/motivators models.

Needs According to Maslow	Herzberg	Needs According to Deci and Ryan	Herzberg Indicators
Self-realization	Intrinsic motivator	Autonomy	Growth, responsibility, challenging work, freedom
Self-Esteem	Intrinsic motivator	Self-Esteem and competence	Recognition, status, progress, achievements
Social	Extrinsic motivator	Relational or affiliation	Quality of interpersonal relations, personnel policy, etc.
Security	Extrinsic motivator		Working conditions, job security
Physiological	Extrinsic motivator		Salary, quality of life

**Table 2:** Descriptive Aggregated Measures.

Instrument	*n*	Min	Max	Mean	*SD*
PWI	75	4.00	10.00	8.05	1.15
Extrinsic Job Satisfaction	75	3.22	9.89	7.59	1.50
Intrinsic Job Satisfaction	75	4.71	10.00	8.18	1.23
General Job Satisfaction	75	4.13	9.94	7.85	1.31
SWFS	75	5.00	10.00	7.96	1.21

**Table 3:** Means according to sex.

Sex	PWI	SWFS	ESLE	ESLI	ESL
Man	7.97	7.95	7.64	8.29	7.92
Woman	8.28	8.04	7.47	7.90	7.66

PWI = General well-being, SWFS = Familiar satisfaction ESLE = Extrinsic job satisfaction, ESLI = Intrinsic job satisfaction, ESL = Total job satisfaction.

**Table 4:** Means according to company size.

How Many People Work in the Company Where You Work?	PWI	SWFS	ESLE	ESLI	ESL
Less than 10	8.88	8.33	8.52	8.71	8.61
Between 10 and 50	7.98	7.87	7.74	8.4	8.03
Between 50 and 100	7.76	8.3	7.63	8.1	7.83
More than 100 people	7.99	7.91	7.36	8	7.64

PWI = General well-being, SWFS = Familiar satisfaction, ESLE = Extrinsic job satisfaction, ESLI = Intrinsic job satisfaction, ESL = Total job satisfaction.

**Table 5:** Means according to marital status.

What is Your Marital Status?	PWI	SWFS	ESLE	ESLI	ESL
Single	8.35	8.3	7.73	8.33	7.99
Married	8.11	8.08	7.7	8.28	7.95
Cohabitant	8.09	7.4	8.11	8.2	8.15
Separated or divorced	7.57	7.5	6.81	7.66	7.18

PWI = General well-being, SWFS = Familiar satisfaction, ESLE = Extrinsic job satisfaction, ESLI = Intrinsic job satisfaction, ESL = Total job satisfaction.

**Table 6:** Correlations between main indexes.

		PWI	SWFS	ESLEA	ESLI	ESL
SWFS	Pearson correlation	0.542 **	1			
	Sig. (bilateral)	0.000				
ESLE	Pearson correlation	0.638 **	0.540 **	1		
	Sig. (bilateral)	0.000	0.000			
ESLI	Pearson correlation	0.539 **	0.552 **	0.798 **	1	
	Sig. (bilateral)	0.000	0.000	0.000		
ESL	Pearson correlation	0.631 **	0.573 **	0.969 **	0.922 **	1
	Sig. (bilateral)	0.000	0.000	0.000	0.000	

** The correlation is significant at the 0.01 level (bilateral). PWI = General well-being, SWFS = Familiar satisfaction, ESLE = Extrinsic job satisfaction, ESLI = Intrinsic job satisfaction, ESL = Total job satisfaction.

**Table 7:** Regression Coefficients Model 1 (Job Satisfaction Scale).

	Non-Standardized Coefficients		Standardized Coefficients	t	Sig.
	B	Standard Error	Beta		
(Constant)	3.464	0.920		3.764	0.000
How satisfied are you with...					
...the physical conditions of their work	0.195	0.078	0.298	2.498	0.015
...the freedom to choose their method of work	0.194	0.090	0.284	2.150	0.036
...their co-workers	0.010 **	0.090	0.015 **	0.110	0.913
...the recognition you get for a job well done	0.284	0.101	0.476	2.813	0.007
...his or her superior or immediate superior(s)	−0.129	0.095	−0.202	−1.364	0.178
...the responsibility that has been assigned to him or her	−0.012 **	0.112	−0.012 **	−0.103	0.919
...your salary	0.001 ***	0.082	0.001 ***	0.007	0.994
...the possibility of using its capabilities	−0.031	0.094	−0.043	−0.333	0.740
...relations between management and the company’s employees	0.161	0.108	0.276	1.487	0.142
...the way in which your company is managed	−0.159	0.084	−0.334	−1.885	0.064
...the attention given to the suggestions it makes	0.008 ***	0.135	0.012 **	0.062	0.951
...your working hours	−0.007 ***	0.055	−0.014 **	−0.118	0.907
...the variety of things you carry out on the job	0.135	0.110	0.175	1.221	0.227
...your job stability	0.087 *	0.069	0.162	1.265	0.211
...the influence it has in the company	0.235	0.111	−0.324	−2.118	0.038
...the workload it has	0.060 *	0.060	0.127	1.008	0.317

*, ** and *** for levels of significance at 10%, 5% and 1%, respectively.

**Table 8:** Regression coefficients Model 2 (Family Satisfaction Scale).

	Non-Standardized Coefficients		Standardized Coefficients	t	Sig.
	B	Standard Error	Beta		
(Constant)	5.154	0.812		6.344	0.000
How satisfied are you with...					
...the degree of closeness among family members	0.220	0.143	0.256	1.532	0.130
...your family’s ability to cope with stress	0.422	0.120	0.597	3.507	0.001
...your family’s capacity for flexibility	−0.084 *	0.122	−0.110	−0.688	0.494
...your family’s ability to share positive experiences	−0.550	0.175	−0.635	−3.148	0.002
...the quality of communication between members of your family	0.148	0.144	0.193	1.024	0.310
...your family’s capacity to resolve conflicts	0.115	0.122	0.157	0.940	0.351
...the time you spend with his family	−0.001 ***	0.068	−0.002 ***	−0.016	0.987
...the way things are discussed as a family	0.066 *	0.120	0.096 *	0.548	0.586
...the relevance of criticisms among family members	0.097 *	0.131	0.127	0.744	0.459
...the concern that the family members have for one another	−0.029 **	0.099	−0.038 **	−0.297	0.768

*, ** and *** for levels of significance at 10%, 5%, and 1%, respectively.

**Table 9:** Regression Coefficients Model 3 (Integrated Model).

	Non-Standardized Coefficients		Standardized Coefficients	t	Sig.
	B	Standard Error	Beta		
(Constant)	3.878	0.667		5.818	0.000
How satisfied are you with...					
...your family’s ability to cope with stress	0.363	0.086	0.512	4.234	0.000
...your family’s ability to share positive experiences	−0.245	0.098	−0.283	−2.502	0.015
...the physical conditions of your work	0.182	0.060	0.278	3.030	0.003
...the freedom to choose their method of work	0.163	0.060	0.240	2.720	0.008
...the recognition you get for a job well done	0.134	0.060	0.224	2.251	0.028
...your job stability	0.116	0.049	0.215	2.375	0.020
...the influence you have in the company	−0.168	0.069	−0.232	−2.442	0.017

**Table 10:** Attributes and good practices mentioned by high-performing managers.

Organizational Features	Leadership Features
Good headquarters and that they support them. Constant work challenges, commensurate with their capabilities. Consistency between what organizations say and what they do. High levels of autonomy. Jobs with purpose (or that make sense to them). Positive peer assessment. Working physical conditions.	Professional respect for subordinates. Promotion of autonomous equipment. Promotion of career development of their subordinates. Recognition of team achievements.

## Data Availability

The datasets generated for this study are available on request to the corresponding author.

## Appendix A

**Table A1:** Descriptive Personal Wellbeing Index.

How Satisfied Are You with...	*n*	Min	Max	Mean	*SD*
...your life in general? (1)	75	0.00	10.00	8.36	1.69
...your standard of living? (2)	75	3.00	10.00	8.56	1.43
...your state of health, in general? (3)	75	5.00	10.00	8.04	1.51
...the achievements you are making in your life? (4)	75	5.00	10.00	8.57	1.27
...your personal relationships? (5)	75	5.00	10.00	8.26	1.33
...how safe and secure you feel? (6)	75	3.00	10.00	7.90	1.63
...with feeling part of your community? (7)	75	2.00	10.00	7.74	1.80
...your safety and security in the future? (8)	75	1.00	10.00	7.26	1.96
PWI	75	4.00	10.00	8.05	1.15

**Table A2:** Descriptive data of the Work Satisfaction Scale.

How Satisfied Are You with…?	*n*	Min	Max	Mean	*SD*
...the physical conditions of their work (1)	75	3.00	10.00	8.26	1.77
...the freedom to choose their method of work (2)	75	1.00	10.00	8.33	1.70
...their co-workers (3)	75	0.00	10.00	7.92	1.79
...the recognition you get for a job well done (4)	75	2.00	10.00	7.52	1.94
...his or her immediate superior(s) (5)	75	1.00	10.00	7.77	1.82
...the responsibility that has been assigned to him or her (6)	75	5.00	10.00	8.70	1.17
...your salary (7)	75	0.00	10.00	7.50	2.04
...the possibility of using its capabilities (8)	75	2.00	10.00	8.24	1.60
...relations between management and the company’s employees (9)	75	2.00	10.00	7.54	1.98
...the way in which your company is managed (10)	75	0.00	10.00	6.76	2.43
...the attention given to the suggestions it makes (11)	75	3.00	10.00	7.76	1.73
...your working hours (12)	75	0.00	10.00	7.70	2.39
...the variety of things you carry out on the job (13)	75	4.00	10.00	8.56	1.50
...their job stability (14)	75	0.00	10.00	8.20	2.14
...the influence it has in the company (15)	75	4.00	10.00	8.20	1.60
...the workload it has (16)	75	0.00	10.00	6.69	2.43
Extrinsic Job Satisfaction	75	3.22	9.89	7.59	1.50
Intrinsic Job Satisfaction	75	4.71	10.00	8.18	1.23
General Job Satisfaction	75	4.13	9.94	7.85	1.31

**Table A3:** Descriptive Family Satisfaction Scale.

How Satisfied Are You with...	*n*	Min	Max	Mean	*SD*
...the degree of closeness among family members (1)	75	5.00	10.00	8.50	1.34
...your family’s ability to cope with stress (2)	75	3.00	10.00	7.73	1.63
...your family’s capacity for flexibility (3)	75	4.00	10.00	7.98	1.52
...your family’s ability to share positive experiences (4)	75	5.00	10.00	8.49	1.33
...the quality of communication between members of your family (5)	75	5.00	10.00	8.00	1.51
...your family’s capacity to resolve conflicts (6)	75	4.00	10.00	8.13	1.57
...the time he spends with his family (7)	75	2.00	10.00	6.86	1.86
...the way they discuss things as a family (8)	75	3.00	10.00	7.62	1.69
...the relevance of criticisms among family members (9)	75	4.00	10.00	7.66	1.50
...the concern that your family members have for one another (10)	75	2.00	10.00	8.68	1.49
SWFS	75	5.00	10.00	7.96	1.21

**Table A4:** Rotated Component Matrix.

	1	2	3	4	5
SWF4sharingpositiveexperiences	0.875				
SWF1closenness	0.844				
SWF3flexibility	0.827				
SWF5communication	0.773				
SWF2copewithstress	0.770				
SWF9relevancecriticism	0.727				
SWF10familyconcern	0.727				
SWF6solvingconflicts	0.689				
SWF8waydiscussthings	0.671				
PWI5relations	0.522		0.488		
PWI1lifesatisfaction	0.480				
ESLI11attentionsuggestion		0.841			
ESLI15influencecompany		0.813			
ESLE9relationmanagersemployes		0.781			
ESLI13amounttvariety		0.716			
ESLE10waymanagement		0.700	0.496		
ESLI8usecapabilty		0.677			
ESLI4recognition		0.627			
ESLE5superiors		0.611			
ESLI6responsabilty		0.551			
ESLE16workload			0.651		
PWI3health			0.651		
PWI7community			0.613		
ESLE3coworkers			0.582		
ESLI2freedom		0.469	0.557		
ESLE1physicalconditions			0.523		
PWI6currentsafety				0.754	
PWI8futuresafety				0.724	
PWI4achievement				0.676	
PWI2leveloflife				0.654	
ESLE14jobsecurity		0.482		0.564	
ESLE12working hours					0.695
SWF7timetogether					0.607
ESLE7salary					0.496
